# Synaptic Plasticity Changes: Hallmark for Neurological and Psychiatric Disorders

**DOI:** 10.1155/2018/9230704

**Published:** 2018-10-23

**Authors:** Giuseppina Martella, Paola Bonsi, Steven W. Johnson, Angelo Quartarone

**Affiliations:** ^1^University of Rome Tor Vergata, Rome, Italy; ^2^IRCCS Fondazione Santa Lucia, Rome, Italy; ^3^Oregon Health & Science University, Portland, USA; ^4^University of Messina, Messina, Italy

Many molecular mechanisms cooperate to produce synaptic plasticity changes. These include alterations in neurotransmitter release and in the effectiveness of neuronal response to those neurotransmitters. Defining and understanding how these mechanisms are involved in neuropathological conditions are a major challenge for neuroscience in the third millennium.

Indeed, synaptic dysfunction is involved in a great number of neurological conditions, such as neurodegenerative diseases (Alzheimer's, Parkinson's, and Huntington's disease), dystonia, levodopa-induced dyskinesia, and ischemia [1–8], as well as neuropsychiatric conditions such as autism, schizophrenia, and major depression [9–15]. Moreover, synaptic plasticity alterations can appear in an early asymptomatic phase of the disease [16, 17].

Therefore, the aim of this special issue is to highlight synaptic plasticity changes as a hallmark of neurological and neuropsychiatric diseases.

In this issue, M. Cantone et al. report altered mechanisms of neural plasticity associated with long-term hand motor deficits in adult patients with intellectual disability. Intellectual disability is commonly associated with impairments in psychomotor skills and abilities of daily living. These authors studied the effects of a two-month program of hand-motor rehabilitation and visual-perceptual treatment in a population of 30 subjects with mild intellectual impairment. The study reports that this group had significantly better motor performance compared to an intellectually impaired group that received conventional rehabilitation. These results suggest that a program that emphasizes visual-perceptual and motor skills may be superior to conventional rehabilitation in improving motor function in the mildly intellectually impaired individuals. From a neuroanatomical perspective, motor learning requires the development and retention of several skills, depending on the structural and functional integrity of the neostriatum and the cerebellum. These brain structures are considered the substrate of learning and memory, both in development and aging and in physiological and pathological conditions [18]. The findings from this report open new exciting windows on the noninvasive rehabilitative interventions targeting the cortical plasticity and neural connectivity.

C, Terranova et al. investigated the mechanism contributing to the selection of voluntary movements in focal hand dystonia, a syndrome characterized by muscle spasms giving rise to involuntary movements and abnormal postures. Significant alterations in synaptic plasticity were described in dystonic animal models as well as in patients [6, 19–22]. In the present work, the authors evaluated the spatial and temporal somatosensory integration by recording somatosensory evoked potentials (SEPs) in controls and patients with focal dystonia. Patients usually present two main abnormalities: greater facilitation and loss of spatial specificity. Here, the authors demonstrated that the inhibitory integration of somatosensory inputs in focal hand dystonia is normal during sensory-motor plasticity.

J. E. Orfila et al. used a model of cardiac arrest and cardiopulmonary resuscitation (CA/CPR) to produce global brain ischemia and assess whether ischemic LTP (a pathological form of synaptic plasticity induced in acute brain slices by oxygen and glucose deprivation) is reproduced *in vivo*. Indeed, the authors found an increased postsynaptic glutamate receptor phosphorylation and function and a preserved ability to depotentiate CA1 synapses. Ischemic LTP was found to occlude physiological LTP, providing a possible target for interventional strategies to improve memory function after cardiac arrest. This is the first study to demonstrate that *in vivo* ischemia causes synaptic alterations that are consistent with ischemic LTP and represent a new model to characterize aberrant forms of synaptic plasticity.

In the last section of this issue, three reviews point to the attention on the molecular mechanisms underlying synaptic plasticity alterations.

U. Shefa et al. discuss the role of diffusible gaseous transmitters (gasotransmitters) in regulating neuronal excitability and plasticity. In this review, the authors summarize recent evidence on the role of hydrogen sulfide, nitric oxide, and carbon monoxide in synaptic plasticity, emphasizing that these gaseous neurotransmitters can play roles in neurological conditions such as schizophrenia, bipolar disorder, major depressive disorder, and Alzheimer's disease. They suggest that rescuing homeostatic levels of gasotransmitters may restore synaptic plasticity and proper neuronal functioning.

P. Olivero et al. perform a thorough analysis of the role of mitochondria in synaptic alterations underlying psychiatric and neurodegenerative disorders. The efficiency of the cellular physiological processes is governed by an appropriate protein localization and function. A molecular network, called the proteostasis network, participates in the intricate mechanisms of synthesis, folding, trafficking, and degradation necessary to ensure the structure and function of proteins. Dysfunction of the proteostatic network affects neuronal plasticity, and the authors discuss the role of some proteins involved in common diseases, in plasticity alteration and neurodegeneration.

The inflammatory cytokines tumor necrosis factor (TNF) and interleukin-1*β* (IL-1*β*) play important physiological roles in LTP and synaptic scaling. However, actions of these cytokines on synaptic plasticity can be altered under conditions of neuroinflammation. F. R. Rizzo et al. provide a timely summary of the important effects of inflammatory cytokines on synaptic plasticity in health and disease and discuss the role of TNF and IL-1*β* in synaptic plasticity under either physiological or inflammatory conditions, with special emphasis on experimental allergic encephalitis and multiple sclerosis.

The contributions collected in the present issue show the importance of correct synaptic adaptations in the maintenance of a physiological state. Overall, these works show that indeed synaptic plasticity changes may represent a hallmark for neurological and psychiatric disorders.
